# Advances in the study of the role of epigenetic mechanisms in plant growth and response to stress

**DOI:** 10.1093/hr/uhag152

**Published:** 2026-04-17

**Authors:** Yanzhu Liu, Jiaqi Wu, Jinhua Yang, Fangfang Xie, Canbin Chen, Xinhua He, Cong Luo, Tianli Guo

**Affiliations:** State Key Laboratory for Conservation and Utilization of Subtropical Agro-Bioresources, Guangxi Key Laboratory for Agro-Environment and Agro-Product Safety, National Demonstration Center for Experimental Plant Science Education, College of Agriculture, Guangxi University, Nanning 530004, Guangxi, China; State Key Laboratory for Conservation and Utilization of Subtropical Agro-Bioresources, Guangxi Key Laboratory for Agro-Environment and Agro-Product Safety, National Demonstration Center for Experimental Plant Science Education, College of Agriculture, Guangxi University, Nanning 530004, Guangxi, China; State Key Laboratory for Conservation and Utilization of Subtropical Agro-Bioresources, Guangxi Key Laboratory for Agro-Environment and Agro-Product Safety, National Demonstration Center for Experimental Plant Science Education, College of Agriculture, Guangxi University, Nanning 530004, Guangxi, China; State Key Laboratory for Conservation and Utilization of Subtropical Agro-Bioresources, Guangxi Key Laboratory for Agro-Environment and Agro-Product Safety, National Demonstration Center for Experimental Plant Science Education, College of Agriculture, Guangxi University, Nanning 530004, Guangxi, China; State Key Laboratory for Conservation and Utilization of Subtropical Agro-Bioresources, Guangxi Key Laboratory for Agro-Environment and Agro-Product Safety, National Demonstration Center for Experimental Plant Science Education, College of Agriculture, Guangxi University, Nanning 530004, Guangxi, China; State Key Laboratory for Conservation and Utilization of Subtropical Agro-Bioresources, Guangxi Key Laboratory for Agro-Environment and Agro-Product Safety, National Demonstration Center for Experimental Plant Science Education, College of Agriculture, Guangxi University, Nanning 530004, Guangxi, China; State Key Laboratory for Conservation and Utilization of Subtropical Agro-Bioresources, Guangxi Key Laboratory for Agro-Environment and Agro-Product Safety, National Demonstration Center for Experimental Plant Science Education, College of Agriculture, Guangxi University, Nanning 530004, Guangxi, China; State Key Laboratory for Conservation and Utilization of Subtropical Agro-Bioresources, Guangxi Key Laboratory for Agro-Environment and Agro-Product Safety, National Demonstration Center for Experimental Plant Science Education, College of Agriculture, Guangxi University, Nanning 530004, Guangxi, China

## Abstract

Plants face the challenge of balancing dynamic environmental stresses with internal developmental demands during growth, necessitating the precise regulation of gene expression networks to optimize resource allocation. Recent studies have shown that epigenetic regulatory systems, which serve as bridges between genotype and phenotype, enable plants to respond rapidly to environmental changes and facilitate adaptive evolution by establishing heritable epigenetic memory. This article systematically elucidates the molecular mechanisms by which core epigenetic processes coordinate plant growth and development with environmental adaptation, with particular emphasis on the transgenerational transmission of epigenetic information, such as DNA modification, RNA modification, histone modification, histone chaperones, chromatin remodeling, and non-coding RNA regulation. It further examines the interrelationships between these mechanisms and how they work in concert to achieve such coordination. It also explores key unresolved scientific questions in this field and proposes future perspectives for further research.

## Introduction

**Figure 1:**
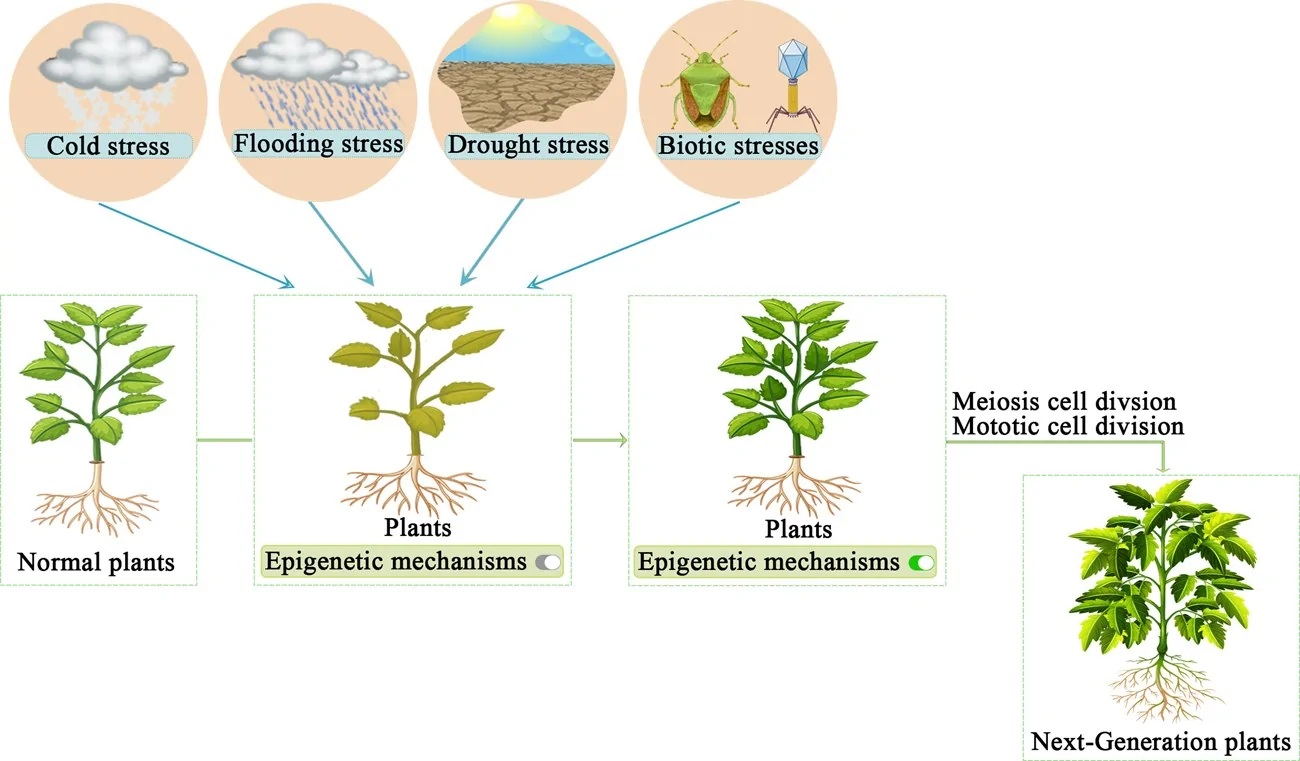
Epigenetic mechanisms transmission diagram: When plants are subjected to biotic and abiotic stresses, epigenetic mechanisms become active, leading to transgenerational inheritance and enhancing their adaptability to variable environments

Epigenetic mechanisms regulate gene expression and influence biological phenotypes without altering the underlying DNA sequence, and these modifications are stably inherited by offspring. Studies have demonstrated that epigenetic mechanisms play a crucial regulatory role in plant growth, development, and responses to environmental stress (Fig. 1) [1]. Based on distinct molecular mechanisms, epigenetic mechanisms include DNA modification, RNA modification, histone modification, histone chaperones, chromatin remodeling, and non-coding RNA (ncRNA) regulation [2, 3]. In the context of intensifying global climate change, frequent extreme weather events pose severe threats to crop growth and reproduction. Because plants cannot rapidly alter their genomic sequences to adapt to environmental changes, epigenetic mechanisms serve as vital strategies for coping with biotic and abiotic stresses. These modifications not only enable rapid responses to environmental challenges but also transmit stress-resistant traits to offspring through transgenerational epigenetic inheritance [3]. Therefore, a comprehensive understanding of epigenetic regulatory mechanisms is essential to enhance crop stress resilience and ensure food security.

## Types and roles of epigenetic mechanisms

### DNA modification

As one of the most thoroughly studied and well-characterized DNA modifications, DNA methylation is the focus of this article. DNA methylation is dynamically regulated by the coordinated actions of methylation and demethylation mechanisms. This process is catalyzed by specific DNA methyltransferases [4]. Demethylation occurs via two pathways: Active demethylation, mediated by repressor of silencing 1 (ROS1)/demeter-like proteins through the base excision repair pathway, and passive demethylation, which results from impaired methyltransferase activity during DNA replication [4, 5].

**Figure 2:**
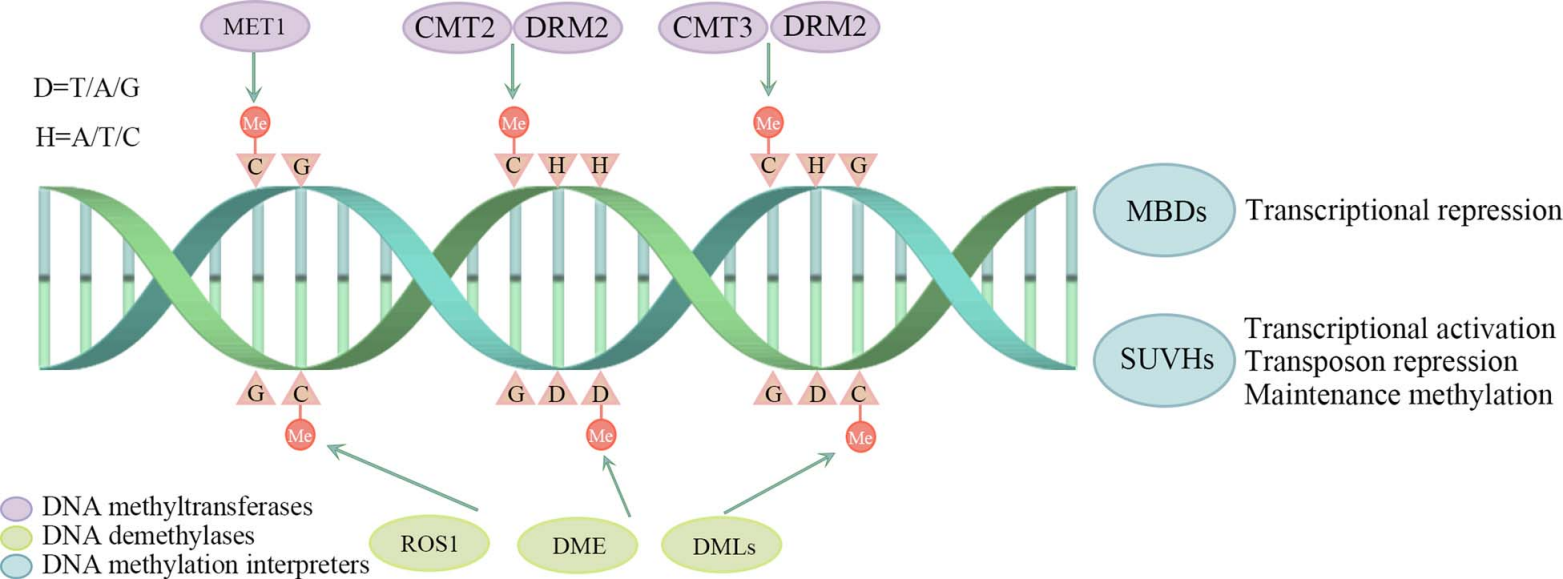
Modification of DNA methylation: Establishment, maintenance, removal and interpretation of methylation in the context of CG, CHG, and CHH sequences

In plants, DNA methylation primarily occurs in three sequence contexts: C-G dinucleotide (CG), C-H-G trinucleotide (CHG), and C-H-H trinucleotide (CHH) (where H = A, T, or C) [4]. Notably, the maintenance mechanisms for methylation in these three sequence contexts exhibit distinct characteristics. Specifically, methylation at CG sites is maintained by DNA methyltransferase 1 (MET1), which plays a crucial role in silencing transposons and repetitive sequences, preventing genomic mutations, and ensuring the transgenerational stability of CG methylation. Methylation at CHG sites is primarily maintained by chromomethylase 3 (CMT3), a member of the plant-specific chromatin methyltransferase (chromomethylase) family. CMT3 recognizes H3K9me2, a repressive histone modification, forming a complementary functional interaction with MET1-mediated CG methylation and domains rearranged methyltransferase 2 (DRM2) mediated CHH methylation to achieve the coordinated transmission of epigenetic information. The methylation of CHH sites depends on DRM2 (a core executor of the RNA-directed DNA methylation (RdDM) pathway) and CMT2. CHH methylation in heterochromatin is maintained by CMT2, whereas CHH methylation in euchromatin or at the edge of long transposable elements (TEs) is mediated by DRM1/2 through the RdDM pathway, DRM2 establishes novel DNA methylation modifications guided by small interfering RNAs (siRNAs) [6]. Studies have revealed that embryo-specific CHH methylation can occur at unmethylated sites in other plant tissues [7]. This multi-tiered methylation regulatory system ensures precise transmission and dynamic regulation of epigenetic information in plants. Canonical methyl-CpG-binding domain proteins are key interpreters of DNA methylation that recognize methylated CG sites and recruit chromatin remodelers, histone deacetylases (HDACs), and histone methyltransferases to repress transcription [8, 9]. DNA methylation is generally considered to be an inhibitory epigenetic marker of gene expression. However, it is important to note that methyl-CpG-binding domain-containing protein 7 in *Arabidopsis* recognizes highly methylated CpG sites and associates with histone acetyltransferase increase in DNA methylation 1 and its related proteins to promote DNA demethylation and prevent gene silencing [9]. Xiao et al. have identified several Su(var)3–9 homologs (SUVHs), including SUVH1, SUVH3, SUVH7, and SUVH8 as DNA methylation interpreters. These proteins bind to DNA methylation monitoring sequence regions through DNA methylation-dependent mechanisms and interact with putative transcriptional activators that contain the DnaJ heat shock protein homolog (DnaJ) domain to promote ROS1 expression. This discovery revealed a critical mechanism: SUVH proteins recognize DNA methylation marks to enhance *ROS1* gene expression, which in turn regulates genome-wide DNA methylation patterns and enhances active demethylation [10] (Fig. 2).

The main types of DNA methylation modifications are summarized in Table 1. Of these, cytosine methylation (5-methylcytosine, 5mC) is the most predominant evolutionarily conserved. This landmark discovery dates back to 1925 when 5mC was first identified in bacteria. With advancements in molecular biology, particularly the elucidation of nucleic acids as genetic carriers and the discovery of DNA double-helix structures, research on DNA methylation has become a central focus in epigenetics. Recent studies have significantly reshaped our understanding of 5mC distribution patterns. Although early theories suggested a random distribution across DNA molecules, new evidence has revealed specific enrichment of 5mC at CpG dinucleotide sequences [11]. First proposed in 1972 for rat brain and liver DNA [12], 5hmC was not officially recognized as a distinct nucleotide until 2009. Tet methylcytosine dioxygenase 1 was the first enzyme to oxidize 5mC to 5hmC [13]. Initially thought to be predominantly present in prokaryotes and certain single-celled eukaryotes [14], subsequent studies have revealed that *N^6^*-methyladenine (6mA) is present in the genomic DNA of viruses, bacteria, protozoa, fungi, and algae, where it functions in plant gene activation and stress responses [15]. Furthermore, *N^4^*-methylcytosine and 5mC play crucial roles in host defense and transcriptional regulation within the bacterial genomes [16].

**Table 1:** Common DNA methylation modifications.

**Type of modification**	**Structure**	**Function**	**Existing in living organisms**
5mC	Cytosine C5 methylation	Gene silencing, transposon inhibition, and genome stability	Plants, animals, fungi
5hmC	5mC oxidation (catalyzed by Tet methylcytosine dioxygenase 1 enzyme)	Demethylated intermediates regulate gene expression (more in mammals, less in plants)	Mainly mammals
6mA	Methylation of adenine *N^6^*	Gene activation, stress response (found in plants, algae, and some animals)	Plants, nematodes, algae, *Drosophila melanogaster*
4-methylcytosine	Cytosine *N^4^* methylation	Bacterial restriction-modification system (rare in plants and eukaryotes)	Mainly bacteria

### DNA methylation regulates plant growth and development via nucleolar dominance and loss

DNA methylation influences growth processes by regulating nucleolar dominance (ND) [17]. Previous studies have indicated that DNA methylation works in conjunction with histone modifications to establish and maintain ND, a mechanism essential for plant development. As a hallmark of epigenetic regulation, ND involves the selective activation of the nucleolar organizing region from one parental source containing 35 to 48S ribosomal DNA sites for ribosomal RNA transcription, whereas the nucleolar organizing region from the other parent remains specifically silenced [18]. This phenomenon serves as an ideal model for studying gene expression regulation and genomic conflicts, and plays a vital role in plant evolution, development, and environmental adaptation.

DNA methylation directly modulates multiple key developmental stages in plants. First, it regulates embryonic development. Studies have shown that the absence of the methyltransferases DRM1 and DRM2 in *Arabidopsis* leads to abnormal development of female gametophytes, whereas MET1 deficiency causes persistent embryonic developmental defects, with phenotypes first appearing during the elongation and asymmetric division stages of fertilized eggs. Loss of CMT3 primarily affects late embryonic development and results in stage-specific phenotypic characteristics [19]. Second, DNA methylation controls the timing of flowering. A previous study has revealed that both spring-related genes, *vernalization* (*VRN1* and *VRN3*), are regulated by histone modifications and that their DNA methylation levels are significantly positively correlated with flowering time. Hypermethylation effectively promotes floral organ development during vernalization in orchardgrass (*Dactylis glomerata*) [20], expanding our understanding of epigenetic regulatory mechanisms underlying flowering. These studies confirm that DNA methylation establishes multilevel epigenetic regulatory networks at critical developmental junctions through precise spatiotemporal dynamics.

### DNA methylation confers stress resistance through multiple mechanisms

Stress can induce mutations at DNA methylation sites in plants [21], and DNA methylation modulates stress responses via a negative feedback mechanism, impacts TEs activation, and functions through other pathways. Several examples are provided below (Table 2). In biotic stress responses, DNA methylation occurs through viral DNA methylation, immune gene demethylation, transposon silencing, transgenerational epigenetic memory, and pathogen counter-defense mechanisms (Table 3).

**Table 2:** Examples of DNA methylation-mediated stress resistance in plants.

**Stress response**		**Theory**	**Genes/transcription factors/proteins**	**references**
Abiotic stress	Illumination	Naturally fluctuating light (FL):DNA methylation may directly regulate gene expression and impact TEs activation	TEs	[22]
	Temperature	High temperature: Under heat stress, reduced DNA methylation at the *ROS1* promoter causes dissociation of the methylation-reading proteins SUVH1 and SUVH3, facilitating the formation of repressive chromatin loops that silence *ROS1* expression. This prevents transposon activation and genomic instability resulting from excessive demethylation; The siRNA-mediated RdDM pathway targets transposons and genes for DNA methylation	*ROS1*; *ZmMOP1*	[23, 24]
		Low temperature: Promoter methylation mediates negative regulation of MYB domain protein 7 (ScMYB7) through transcriptional silencing	ScMYB7	[25]
	Water	Drought: Repression of the *CUC2 domain-containing transcription factor 111* (*ZmNAC111*) expression by the RdDM pathway	*ZmNAC111*	[26]
	Salt/Osmostress	Salt: Stress induces changes at DNA methylation sites but also triggers *GhDMT7*-mediated negative feedback mechanisms that regulate stress responses via DNA methylation; Whole-genome methylation maintains positive regulation of gene expression	*GhDMT7*	[27, 28]
	Nutrition	Phosphorus (P): P starvation reversibly induced DNA methylation in transposable elements close to highly induced genes in plants	/	[29]
	Heavy metal	Aluminum (Al): The multiretrotransposon-like insertion and its degree of DNA methylation influence *hordeum vulgare aluminum-activated citrate transporter 1* (*HvAACT1*) expression to withstand Al toxicity in acid soils	*HvAACT1*	[30]
Biotic stress	Bacteria	*Pseudomonas syringae* pv. tomato DC3000 (Pst DC3000): Elongator acetyltransferase complex subunit 2 (elp2) causes dramatic genome-wide DNA methylation changes, including increased total number of methylcytosines	*AtELP2*	[31]

**Table 3:** Classification of DNA methylation regulatory biotic stress pathways.

**Channel**	**Molecular mechanism**	** Effect**	**Case**	**References**
Viral DNA methylation	RdDM pathway (CLCuMuVV2-AGO4-DRM2)	Inhibit viral replication	Geminiviral CHH methylation	[32]
Immune gene demethylation	ROS1 removes defense gene methylation	Activate the SA/JA pathway	*Arabidopsis* and *Nicotiana* demethylation	[33]
Transposon silencing	Mechanisms, such as DNA methylation, histone modification, and RNA silencing, form a comprehensive genomic defense system	Prevent genetic damage	*Arabidopsis*	[34]
Transgenerational memory	Methylated variants are passed on by gametes	Resistant transgenerational inheritance	Corn backcrossing and hybrid breeding maintain induced epigenetic states for intergenerational inheritance; Steeped stress was transmitted through female gametes in *Arabidopsis*	[35, 36]
Pathogen counter defense	Beet severe curly top virus (BSCTV) infection induces *VIM1* expression, which epigenetically activates viral gene transcription	Virus enhances its own replication and infection	*Arabidopsis* infected with BSCTV	[37]

### RNA modification

**Figure 3:**
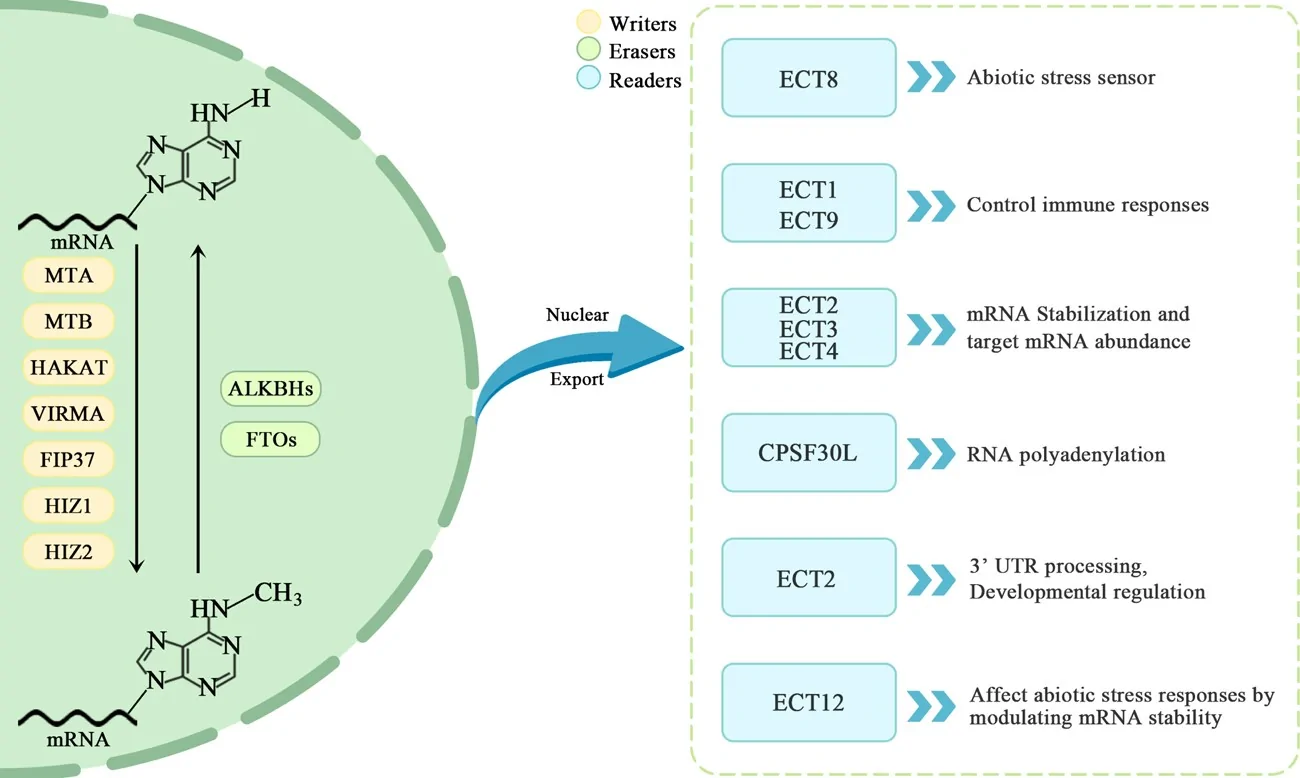
The m^6^A modification pathway in plants: Beyond dynamic regulation to nuclear export and functional diversification

More than 160 RNA modifications have been identified [38]. Common RNA modifications include *N*^*6*^-methyladenosine (m^6^A), acetylation (e.g. *N*^4^-acetylcytidine, ac^4^C), *N*^7^-methylguanosine (m^7^G), 5-methylcytidine (m^5^C), *N*^1^-methyladenosine (m^1^A), inosine (I), *N*^*6*^, 2′-O-dimethyladenosine (m^6^Am), and pseudouridine (Ψ), among others [38, 39]. m^6^A methylation modification is the most abundant internal modification in messenger RNA (mRNA) across all eukaryotes [40]. Therefore, it is one of the RNA modifications that are emphasized in this article. Related studies have indicated that m^6^A modification is a dynamic and reversible process regulated by methyltransferases (writers), demethylases (erasers), and binding proteins (readers) [38] (Fig. 3).

### RNA methylation regulates plant growth and development

RNA methylation modifications, such as m^6^A, play a critical regulatory role in plant nutrient growth, and reproductive development responses [41]. Studies have demonstrated that the absence of key subunits of the methyltransferase complex results in severe developmental defects, including embryonic lethality in *Arabidopsis* [42] and maize [43]. For example, embryos of the *Arabidopsis* SALK_074069 homozygous mutant arrested at the spherical stage, with m^6^A modification completely undetectable in their poly(A) RNA. Further research has shown that the lack of mRNA adenosine methylase A (MTA) mediated m^6^A modification disrupts the expression of genes essential for embryonic development and causes abnormal regulation of signaling pathways, thereby impairing normal embryogenesis [44]. Moreover, modifications to RNA methylation are crucial for the regulation of plant flowering. FIONA1 is a unique methyltransferase i.e. homologous to human METTL16 but has plant-specific functions. A study has shown that FIONA1 does not regulate the methylation or expression of *Arabidopsis* SAM synthase transcripts. Instead, it directly binds to and methylates the transcripts of flowering-related genes, such as *cryptochrome 2*, *flowering locus C (FLC*), and *constans*. This methylation influences their expression levels and ultimately regulates the expression of the flowering hormone-encoding gene *flowering locus T* (*FT*) [45]. In addition to methyltransferases, RNA-binding proteins participate in flowering regulation. For example, the RNA-binding protein flowering locus K homology domain, which acts as a reader of m^6^A modifications, targets and binds to *FLC* transcripts, influencing flowering transitions by regulating their stability and splicing processes [46]. During fruit ripening, MTA-mediated m^6^A methylation promotes the mRNA stability of *9-Cis-Epoxycarotenoid dioxygenase 5* and *ABA responsive element binding protein 1* in the ABA biosynthesis and signalling pathways, whilst simultaneously promoting the translation of ABA receptor ABAR/magnesium chelatase H subunit (*ABAR*); That is, m^6^A targets the ABA pathway to regulate the ripening of non-climacteric strawberry fruits [47].

Furthermore, the integration of post-transcriptional modifications and polyadenylation signaling is a unique feature of plants. The YTH (YT521-B homology) domain of the reader protein cleavage and polyadenylation specificity factor 30-long (CPSF30-L) plays a guiding role by binding to m^6^A-modified UGUA motifs, thereby regulating alternative polyadenylation [48]. This process participates in flowering time regulation and nutrient absorption, enhances plant stress resistance, and influences plant interactions with fungi and viruses [49].

### RNA methylation modulates plant stress resistance by regulating mRNA stability, alternative polyadenylation, and translation efficiency

In plants, m^6^A modification is dynamically responsive to various abiotic and biotic stresses, including salt, drought, viral pathogens and fungal diseases. Multiple studies have demonstrated that stress induces m^6^A deposition on specific stress-responsive transcripts, which in turn modulates their stability, alternative polyadenylation, and translation. Common examples of plant responses to biotic and abiotic stresses mediated by RNA modifications such as m^6^A methylation are presented in Table 4.

**Table 4:** Examples of m^6^A methylation-mediated stress resistance in plants.

**Stress response**		**Theory**	**Genes/transcription factors/proteins**	**References**
Abiotic stress	Illumination	Dark: The level of m^6^A increases during dark-induced leaf senescence and disrupts the stability of senescence-related genes *senescence-associated gene 21* (*SAG21*) and *non-yellowing 1* (*NYE1*), thereby preventing premature senescence	*AtMTA*	[50]
	Temperature	High temperature: AtALKBH10B modulates gene expression variability under high temperature, affecting reproductive thermotolerance	AtALKBH10B	[51]
		Low temperature: Heat shock protein 70 (HSP70) specifically interacts with m^6^A-modified transcripts, potentially enhancing the stability and translation of key mRNAs during stress adaptation; By inducing the expression of cytosolic m^6^A methyltransferase complex (such as fkbp12-interacting protein 37: FIP37), the m^6^A labeling of mRNA is increased, and the translation of thylakoid structure, photosynthetic system, and related proteins is regulated to stabilize photosynthesis against cold	MiHSP70; AtFIP37	[52, 53]
	Water	Drought: Evolutionarily conserved c-terminal region (AtECT12 and AtECT8) affect the stability of mRNA involved in drought stress response; m^6^A-mediated sequestration of PYL7 by AtECT8 in stress granules negatively regulates abscisic acid (ABA) perception, thereby enabling prompt feedback regulation of ABA signalling to prevent plant cell overreaction to environmental stresses; ALKBH10B mediated m^6^A demethylation improves drought tolerance by affecting the stability of mRNA in *Arabidopsis*; GhALKBH10B-mediated removal of m^6^A methylation from transcripts in ABA and calcium signaling pathways negatively regulates cotton drought response; Overexpression of *YTH domain-containing RNA-binding protein 2* (*MhYTP2*) enhances apple water-use efficiency by activating ABA and ethylene signaling	AtECT12; AtECT8; AtALKBH10B; GhALKBH10B; *MhYTP2*	[54–59]
	Oxygen	Oxygen stress: The *AtMTA* mutant was more sensitive to copper-induced oxidative stress; *MhYTP1* and *MhYTP2* act as positive regulators in the hypoxia response, and overexpression plants are more resistant to waterlogging	*AtMTA*; *MhYTP2*	[60, 61]
	Salt/Osmostress	Salt: m^6^A methyltransferase complex (SlMTC) interacts with SlMTA in tomato to influence seed size and the plant response to salt stress; ALKBH10B mediates m^6^A demethylation affects seed germination under salt stress and ABA presence; ECT8 confers salt tolerance by binding to m^6^A on stress-responsive mRNAs in the cytoplasm, enhancing their stability and consequently increasing the accumulation of corresponding proteins; ECT8 interacts with the decapping protein DECAPPING5 in P-bodies, accelerating the degradation of m^6^A-modified mRNAs, especially those encoding negative regulators of salt stress	SlMTC; AtALKBH10B; AtECT8	[55, 62–64]
	Nutrition	Nitrate (N): CPSF30-L regulates the expression of the nitrate transporter gene *NRT1* in *Arabidopsis*; MhYTP2 enhances apple resistance to N deficiency by affecting the stability of bound mRNA	CPSF30-L; MhYTP2	[65, 66]
	Heavy metal	Cadmium (Cd): In switchgrass, Cd stress alters m^6^A modification of basic helix–loop–helix transcription factor bHLH family transcripts, which is associated with changes in their mRNA stability	bHLH113	[67]
Biotic stress	virus	*Alfalfa mosaic* virus: AtALKBH9B-mediated m^6^A demodification on the viral genome regulates viral infection	AtALKBH9B	[68]
		Wheat yellow mosaic virus disease (WYMV): In plant-virus interactions, *TaFIP37-1* and *TaALKBH29B* may be involved in distinct m^6^A modifications between WYMV-infected resistant (WRV) and sensitive (WSV) wheat varieties, however, further experiments are needed to confirm this finding	*TaFIP37-1* and *TaALKBH29B*	[69]
		RNA virus *Cucumber mosaic virus* (CMV): ECT8 acts as an m^6^A reader to destabilize viral RNAs, while CMV-2b counteracts this by binding the m^6^A methyltransferase complex (MTB/HAKAI) to suppress m^6^A deposition, thereby evading host antiviral defense	AtECT8	[70]
		*Pepino mosaic* virus (PepMV): PepMV RdRP interacts with SlBeclin1 to induce autophagic degradation of SlHAKAI, a core component of the m^6^A methyltransferase complex, thereby suppressing m^6^A-mediated plant defense	SlHAKAI	[71]
	Fungus	Powdery mildew disease: MhYTP2 regulates *MLO gene family member 19* (*MdMLO19*) mRNA stability and antioxidant genes translation efficiency, conferring powdery mildew resistance in apple	MhYTP2	[72]
		Glomerella leaf spot: MhYTP2 negatively modulates apple Glomerella leaf spot resistance by binding to and degrading *resistance gene analogue 2-like* (*MdRGA2L*) mRNA	MhYTP2	[73]

### RNA acetylation is involved in many developmental processes

Beyond the well-established RNA methylation, RNA acetylation has recently emerged as a novel layer of epitranscriptomic regulation in plants, implicated in diverse developmental processes. Among these modifications, *N*^*4*^-acetylcytidine (ac^4^C) is currently the predominant known acetylation modification on eukaryotic mRNA. It is catalyzed by conserved N-acetyltransferase 10 (NAT10)/N-acetyltransferases for cytidine in RNA (ACYR) proteins across species. The ac^4^C modification is widely distributed in both nuclear and plastid transcripts, with enrichment in coding regions [74]. Functionally, it orchestrates RNA metabolism through multiple mechanisms: Enhancing mRNA stability to prolong transcript half-life, promoting ribosome binding to improve translational efficiency, and influencing alternative splicing to diversify gene expression products [75, 76].

This modification plays pivotal roles in plant growth and development. Notably, ac^4^C is highly enriched in plastid transcripts, especially those of photosystem-related genes, and contributes to chloroplast structural homeostasis by regulating nuclear-encoded chloroplast development genes [76]. In rice, OsNAT10-mediated ac^4^C regulates shoot development and panicle number [74]. In *Arabidopsis*, the essentiality of ac^4^C is underscored by the embryonic lethality of *AtACYR1 AtACYR2* double mutants, highlighting its critical role in embryogenesis and vegetative growth [39]. NAT10-mediated ac^4^C modification of *flowering locus M* (*FLM*) mRNA directly controls its alternative splicing, ensuring an optimal balance between *FLM-β* and *FLM-δ* splicing variants to promote flowering under low temperatures [76]. During tomato fruit ripening, dynamic changes in ac^4^C modification are observed. It participates in multiple ripening processes by regulating genes involved in *ethylene synthesis*, signaling (ethylene receptor, ethylene response factor), cell wall metabolism (polygalacturonase: *PG*, cellulose synthase A: *CesA*, β-Galactosidase: *β-Gal*), and flavor compound synthesis (glutamate decarboxylase: *GAD*, sucrose synthase: *SUS*) [77].

### RNA acetylation regulates plant stress responses

In recent years, research into the regulatory roles of RNA acetylation in plant responses to stresses has been progressively carried out. In terms of abiotic stress, the level of ac^4^C modification on plant mRNA is significantly elevated under high light stress. ACYR/NAT10-mediated ac^4^C modification maintains the stability of the photosystem, enhances non-photochemical quenching capacity, and reduces photosystem damage under high light by improving the translational efficiency of genes in the light-harvesting complex II B proteins family [74]. In terms of biotic stress, during rice resistance to *Magnaporthe oryzae* infection, the expression of *OsNAT10*/*ACYR* is induced, and the ac^4^C modification mediated by it is enriched in the transcripts of *allene oxide cyclase* (a key gene in the JA synthesis pathway) and *OsERF77* (a transcription factor gene). By improving the translational efficiency of these genes, ac^4^C modification promotes JA accumulation and the expression of downstream defense genes *(allene oxide synthase 2*: *OsAOS2*, *lipoxygenase 3*: *OsLOX3*), thereby enhancing plant immune responses [78]. These findings provide novel insights into dissecting the epigenetic mechanisms underlying plant stress resistance.

### Histone modification

**Figure 4:**
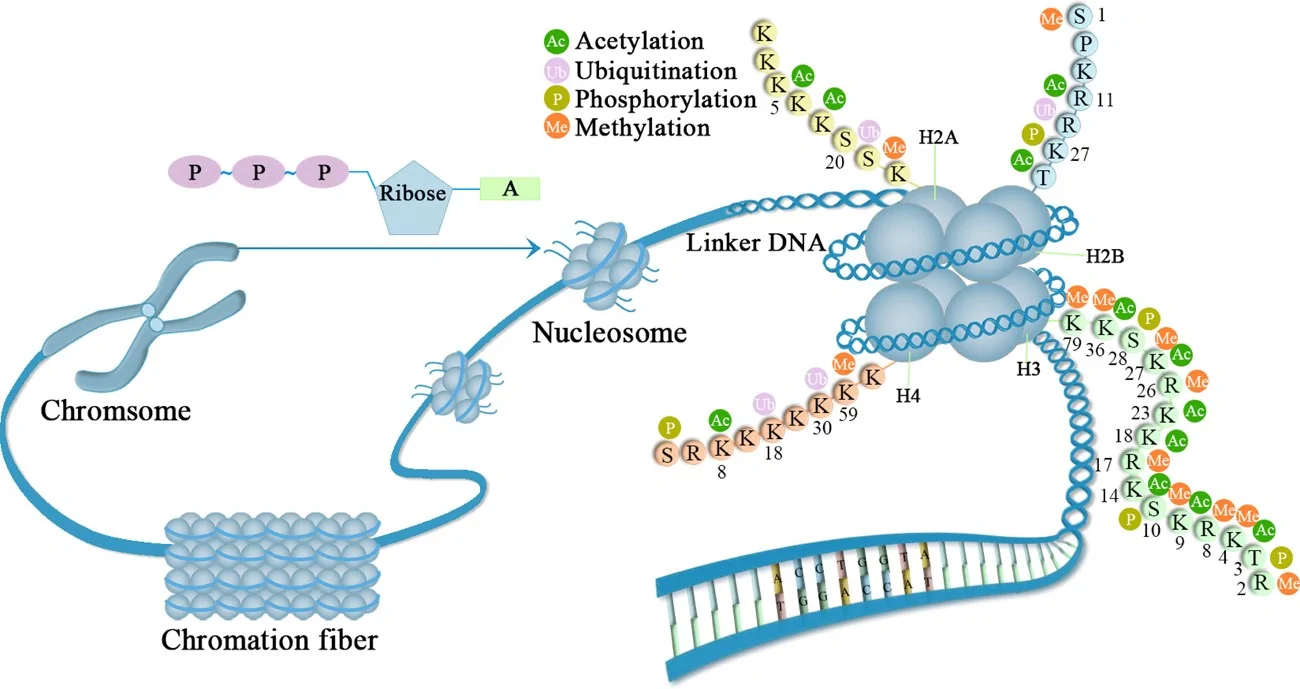
Relationship between histones and chromatin: Hierarchical packaging and epigenetic regulation

In eukaryotes, DNA forms the basic structural unit of chromatin, the nucleosome, by winding around the histone octamers. Each nucleosome core particle is composed of paired H2A, H2B, H3, and H4 histones [79] (Fig. 4). Notably, the nucleosome structure is not static, but exhibits significant plasticity and dynamism [80]. In addition to canonical histones, cells selectively incorporate histone variants (e.g. Histone H2A Variant Z: H2A.Z and Histone H3 Variant 3: H3.3) to confer unique chromatin characteristics to specific genomic regions [81].

Histone modifications are key mechanisms of epigenetic regulation, primarily involving classical modifications, such as acetylation, methylation, ubiquitination, and phosphorylation [82].

**Figure 5:**
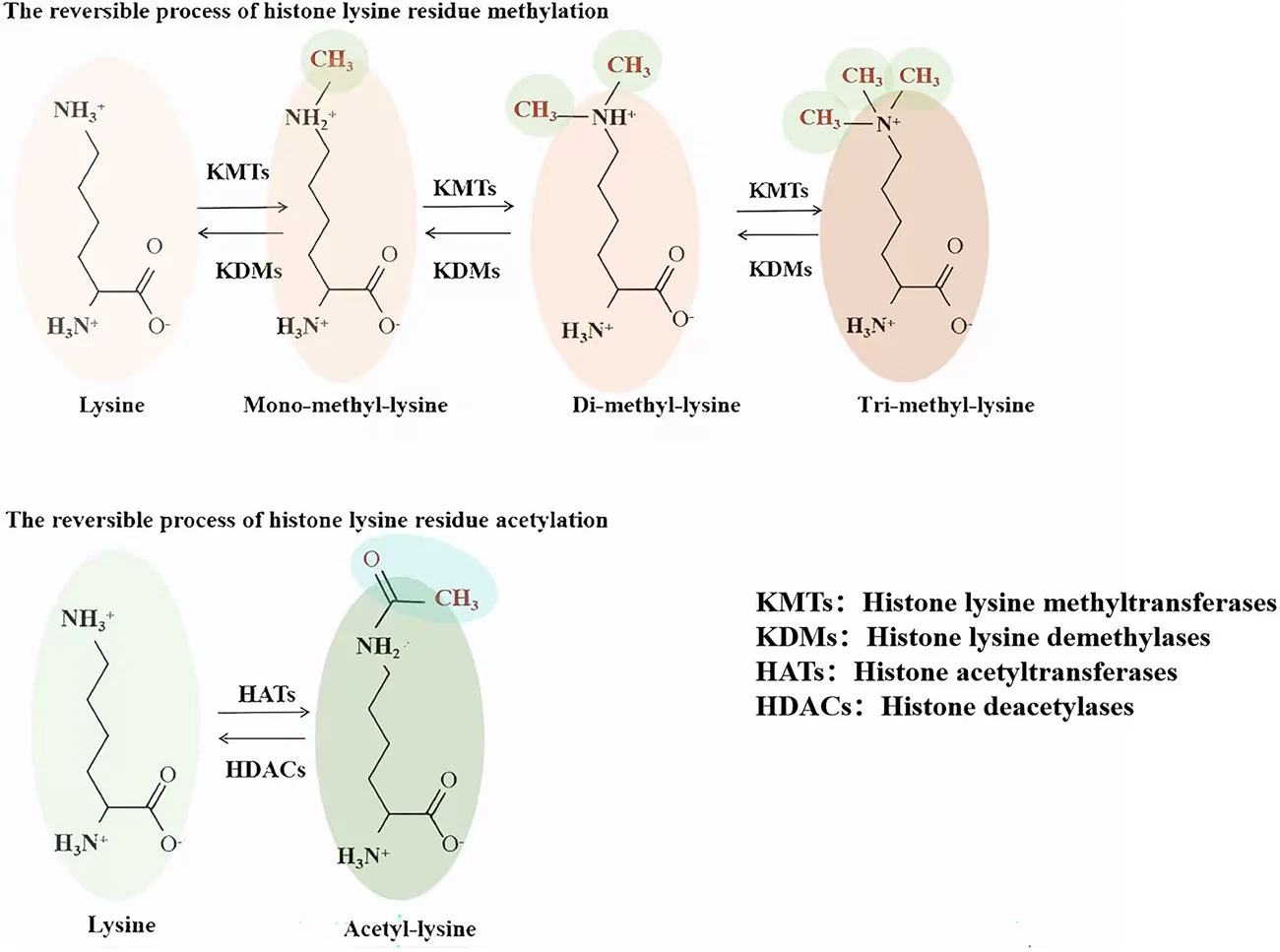
Reversible processes of lysine residue methylation and acetylation in histones: KMTs and KDMs dynamically regulate histone lysine residue methylation; HATs and HDACs dynamically regulate histone lysine residue acetylation

**Figure 6:**
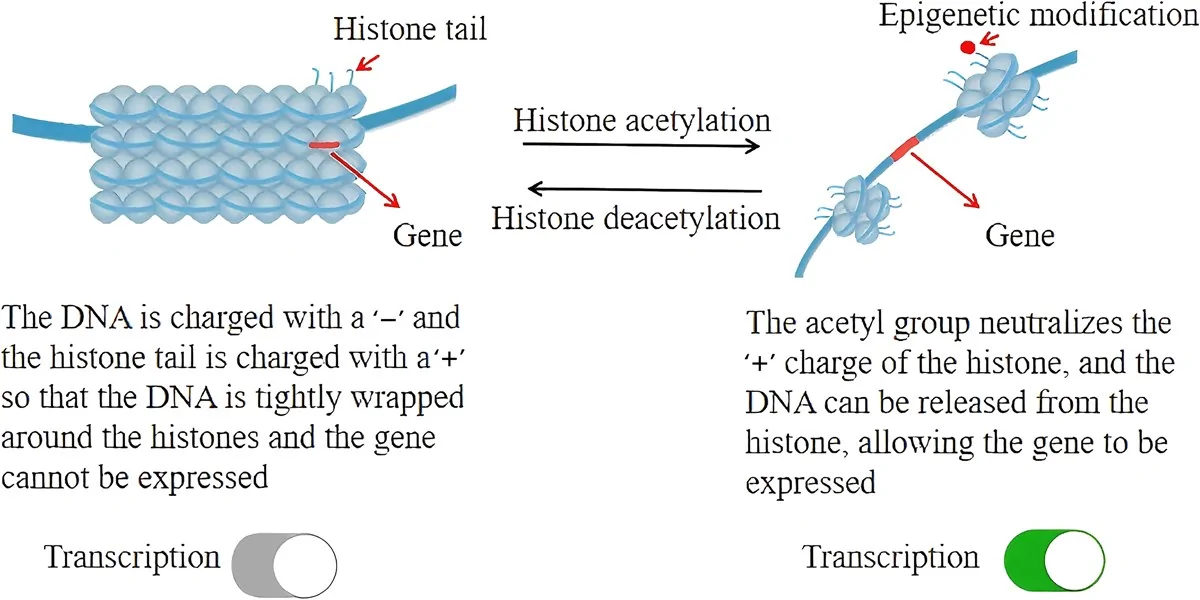
Neutralization of histone LysAc with a positive charge enables gene expression: Charge-neutralization model highlights the fundamental role of histone acetylation as a molecular switch, governing the transition between transcriptionally silent and active states of the genome

In recent years, with advances in research, a series of novel histone modifications have been identified, including crotonylation, which is enriched at transcription start sites and enhancer regions; Malonylation, which plays a role in metabolic regulation; And lactylation, which mediates immune responses [83, 84]. Among these, histone H3 lysine methylation at positions 4, 9, 27, and 36, and lysine acetylation (LysAc) at positions 9 and 27, are the most extensively studied. Histone lysine methylation and acetylation are both dynamic and reversible modification processes (Fig. 5) that play crucial roles in plant stress responses [85, 86]. However, these two modifications differ significantly in their mechanisms of action; Lysine methylation does not alter the amino acid charge and primarily functions by recruiting recognition proteins through the methyl side chain, whereas LysAc neutralizes positive charges, substantially reducing the affinity between histones and DNA [87], thereby facilitating gene expression (Fig. 6).

### Histone methylation modifications at distinct loci exert unique biological functions

Take the most extensively studied loci 4, 9, 27, and 36 as examples. H3K4 methylation is predominantly enriched in gene promoter regions and is a characteristic of activating modifications. These modifications enhance gene expression by recruiting methyl-binding proteins and transcriptional activators. Studies have shown that H3K4 trimethylation regulates plant development and environmental adaptability. Moreover, H3K4 trimethylation and the COMPASS-like complex are essential for *FLC* gene expression and transition from vegetative to reproductive growth in plants [88]. By contrast, H3K9 methylation primarily contributes to heterochromatin formation and gene silencing. This modification plays a critical role in chromosome condensation, pairing, and transposon silencing [89]. The methyltransferase SET domain group 714 specifically targets chromatin regions to catalyze H3K9 dimethylation, thereby regulating growth and development [90]. In *Arabidopsis*, the target of rapamycin signaling pathway suppresses the expression of stress response genes by maintaining global levels of H3K27 trimethylation (H3K27me3). This process depends on the synergistic action of curly leaf (CLF) methyltransferase and the like heterochromatin protein 1 [91]. H3K36 methylation is predominantly distributed in the gene-coding regions and plays a role in transcription termination, mRNA processing, and chromatin stability. Genes marked with H3K36me exhibit an open chromatin state but display very low transcriptional activity. Genetic evidence indicates that the methyltransferase SDG8 maintains H3K36me levels by inhibiting the activity of SDG4 in short and intronless genes, which is essential for ensuring plant fertility [92].

### Histone methylation confers stress resistance through multiple strategies

Under stress conditions, plants precisely regulate the histone methylation modification profile. Activating methylation (H3K4me3, H3K36me3) is rapidly enriched at genes in positive regulatory pathways such as antioxidant defense, osmotic adjustment, and hormone signaling to initiate defense responses, whereas repressive methylation (H3K27me3, H3K9me2) selectively targets and silences non-essential genes to reduce resource consumption [93]. In addition, histone methylation forms a synergistic network with chromatin remodeling complexes and the DNA methylation system [94], enabling cascade amplification and precise transduction of stress signals (Table 5).

**Table 5:** Examples of histone methylation-mediated stress resistance in plants.

**Stress response**		**Theory**	**Genes/transcription factors/proteins**	**References**
Abiotic stress	Illumination	Ultraviolet-C Radiation (UV-C): The umonji domain-containing protein 27 (JMJ27) forms a complex with DNA damage-binding protein 2 (DDB2), and targets the H3K9me2-marked regions upon UV-C exposure. JMJ27 promotes heterochromatin relaxation, thereby enabling DDB2 to access photolesions for further processing by the global genome repair machinery	JMJ27	[95]
	Temperature	High temperature: Histone H3K4 methyltransferases SDG25 and ATX1 maintain heat-stress gene expression during recovery in *Arabidopsis* by regulating H3K4me3 and antagonizing DNA methylation	SDG25 and ATX1	[96]
		Low temperature: Cold-induced deposition of H3K4me3 and H3K27me3 is dependent on ATX1 and CLF, respectively	H3K4me3–H3K27me3	[97]
	Water	Drought: The demethylase JMJ710 directly binds to the chromatin of *MYB48-1*, regulates the level of H3K36me2, and thereby inhibits *MYB48-1* expression; Under drought stress, *SsJMJ4* transcript and protein levels decrease, leading to increased H3K27me3 enrichment and transcriptional repression of *WRKY54* and *WRKY70* homologs in sugarcane	*OsJMJ710*; *SsJMJ4*	[98, 99]
	Salt/Osmostress	Salt: The transcription of the key salt-response regulator radialis-like sant (RSM1), a MYB-related transcription factor involved in ABA-mediated salt stress signaling, was potentially regulated by bivalent H3K4me3-H3K27me3 modifications; Soybean Alfin1-like protein GmPHD6 enhances salt stress tolerance by recognizing low-methylated H3K4 marks and binding to the promoter regions of target genes	H3K4me3-H3K27me3; GmPHD6	[100, 101]
	Nutrition	N: The *Arabidopsis* histone methyltransferase SDG8 mediates genome-wide changes in H3K36 methylation at specific genomic loci functionally relevant to nitrate treatments	AtSDG8	[102]
	Heavy metal	Fe: The nitrate regulatory gene 2/ELF8 complex regulates iron homeostasis by modulating H3K4me3 deposition at target loci such as *GRF11*	*GRF11*	[103]
		Cd: Overexpression of the histone demethylase gene *SlJMJ524* confers Cd tolerance by modulating the expression of genes related to metal transport, the GSH-PC complex pathway, and flavonoid synthesis	*SlJMJ524*	[104]
Biotic stress	Fungus	*Alternaria alternata*: *Alternaria alternata Set* (*AaSet1* and *AaSet2*) are required for the transcriptional regulation of numerous genes involved in ROS detoxification, cell wall degradation, secondary metabolites biosynthesis, and RNA processing in this notorious pathogen	*AaSet1* and *AaSet2*	[105]

### Histone lysine acetylation possesses multiple biological functions

Histone LysAc is a highly conserved post-translational modification (PTM), with the majority of acetylation events occurring on non-chromosomal proteins [106]. Both histone and non-histone proteins in prokaryotes and eukaryotes are subject to LysAc [107]. To date, histone LysAc sites have been identified in various plant species, including *Arabidopsis* [108], rice [109], strawberry [110], tea [111], maize [112], pea [113], and *Rosa roxburghii* Tratt [114]. This modification plays a critical role in plant growth and development by participating in multiple biological processes. For instance, it is involved in photosynthesis and the regulation of carbon assimilation, contributing to the transition of etiolated seedlings from skotomorphogenesis to photomorphogenesis [112]. It also interacts synergistically with protein phosphorylation to influence circadian rhythms in *Arabidopsis* [115]. Furthermore, LysAc modulates key metabolic pathways such as glycolysis, the tricarboxylic acid cycle, and the pentose phosphate pathway [107, 116]. In reproductive development, it contributes to meiosis and anther development in rice and can mediate cytoplasmic male sterility by affecting energy metabolism and pollen development [117]. In perennial plants, histone LysAc regulates germination signaling pathways [118, 119]. It is also involved in hormone-mediated regulation of sucrose metabolism, thereby influencing fruit quality in *Rosa roxburghii* Tratt [114]. Collectively, histone LysAc is engaged in numerous processes that govern plant growth and development, exerting a profound influence on cell fate determination and organ morphogenesis. Nevertheless, many aspects of its regulatory functions and associated networks remain unexplored and warrant further investigation.

### Histone lysine acetylase unlocks multi-pathway stress resistance, bolstering plant homeostasis

Extensive research indicates that histone LysAc regulates abiotic stress by mediating signal transduction, respiration and energy metabolism, protein synthesis, and amino acid metabolism. While in biotic stress responses, it primarily operates through remodelling transcriptional networks, regulating metabolic processes, and modulating immune responses [107] (Table 6).

**Table 6:** Examples of histone LysAc-mediated stress resistance in plants.

**Stress response**		**Theory**	**Genes/transcription factors/proteins**	**References**
Abiotic stress	Illumination	Dark: Lysine 2-hydroxyisobutyrylation (Khib) and H3K23ac act in concert to dynamically modulate the expression of key enzyme-encoding genes, thus fine-tuning plant responses to dark-induced starvation	Khib and H3K23ac	[120]
	Temperature	High temperature: High temperature induces significant changes in the acetylation patterns of photosynthesis-related proteins in grape	/	[121]
		Low temperature: The acetylation levels of lysine residues at positions 27 and 36 on histone H3 are significantly elevated under cold stress	/	[122]
	Water	Flooding: LysAc of carbon metabolism and photosynthetic enzymes promotes metabolic rebalancing, enabling *Kandelia candel* to withstand daily flooding	/	[123]
		Drought: Both SuSy and AGPase, two key enzymes, are subject to acetylation, with their acetylation modifications playing a crucial role in starch biosynthesis and yield formation under water deficit conditions	/	[124]
	Salt/Osmostress	Salt: WRKY53 enhances salt stress resistance by downregulating HDA9, a HDAC that represses stress-responsive transcription	HDA9 and WRKY53;	[125]
	Nutrition	N: Reversible acetylation of antenna proteins is associated with the maintenance of photosynthetic rate in tea leaves following nitrogen application	/	[111]
	Heavy metal	Cd: Increased levels of histone acetylation can reduce cadmium-induced reactive oxygen species accumulation, thereby alleviating cell cycle arrest and DNA damage	/	[126]
Biotic stress	Bacteria	Paulownia witches' broom disease: LysAc affects the function of photosynthesis-related proteins, as evidenced by the identification of differentially acetylated proteins in response to phytoplasma infection	/	[127]
	Fungus	*Cochliobolus carbonum* 1: Cochliobolus carbonum produces HC-toxin, which inhibits host HDACs, leading to histone hyperacetylation. This hyperacetylation reprograms transcriptional networks, resulting in an ineffective defense response that facilitates pathogen virulence	/	[128]
	virus	Chinese wheat mosaic virus: LysAc plays a key role in regulating photosynthesis during viral infection, as evidenced by the significantly increased acetylation levels of photosystem II complex, photosystem I subunits, and ATP synthesis-related chloroplast proteins	/	[129]

### Histone chaperones

In recent years, it has also been found that histone chaperones are essential key epigenetic regulatory factors for plant growth and stress adaptation. Histone chaperones are a conserved family of ATPase-inactive proteins that specifically recognize and bind histones (either H2A/H2B dimers or H3/H4 tetramers) [130, 131]. Their core functions include mediating nucleosome assembly and disassembly, facilitating histone nuclear import and storage, and preventing aberrant histone-DNA interactions. These activities are fundamental to ensuring the dynamic regulation of chromatin [132]. In plants, histone chaperones encompass multiple subfamilies, such as nucleosome assembly protein 1 (NAP1) [132], nap1-related protein [133], anti-silencing function 1 (ASF1) [134], histone regulator A (HIRA) [135], facilitates chromatin transcription (FACT) [136], chromatin assembly factor 1 (CAF-1) [137], and nucleophosmin (NPM) [138], each of which binds to distinct histone variants to form specialized functional modules.

### Histone chaperones orchestrate plant growth, development, and stress responses

Histone chaperones play a pivotal role in plant growth and development, and participate in cell fate determination and organogenesis by regulating chromatin states. For instance, *Arabidopsis* NRP1, as an H2A/H2B histone chaperone, directly interacts with the transcription factor werewolf (WER) and is recruited to the promoter region of the *glabra2* (*GL2)* gene; It activates *GL2* transcription by mediating histone eviction and nucleosome dissociation, thereby regulating the differentiation of root epidermal cells into non-hair cells. Its N-terminal α-helix-mediated dimerization and C-terminal acidic domain are critical for maintaining its interactions with histones and WER, as well as for its functional execution [133]. NPM is classified as an H2A-H2B histone chaperones, but it remains poorly studied in plants [138]. FACT promotes transcript elongation by destabilizing nucleosomes during RNA polymerase II transcription [136]. CAF-1 stabilizes heterochromatin, thereby silencing genes embedded within it [137]. In temperature-responsive morphogenesis, the histone chaperones ASF1a/b interact with ELF7, a subunit of the RNA polymerase II-associated factor 1 complex (PAF1c). They mediate the deposition of the histone variant H3.3 via the HIRA complex, cooperate with the inositol requiring 80 (INO80) chromatin remodeling complex to drive H2A.Z eviction, enhance the transcriptional elongation efficiency of RNA polymerase II, and regulate the expression of auxin-related genes such as *yucca8* and *indole-3-acetic acid 19*, thus promoting the elongation of hypocotyls and petioles in *Arabidopsis* under high temperature conditions [139].

In stress responses, histone chaperones regulate genomic stability and the expression of stress-related genes by cooperating with chromatin remodelers, DNA repair enzymes and other factors (Table 7).

**Table 7:** Examples of histone chaperones mediated stress resistance in plants.

**Stress response**		**Theory**	**Genes/transcription factors/proteins**	**References**
Abiotic stress	Illumination	Light: The *Arabidopsis* histone chaperone FACT promotes the expression of anthocyanin biosynthetic genes under light stress, thereby contributing to stress-induced anthocyanin accumulation	FACT	[136]
	Temperature	High temperature: ASF1, a histone chaperone for H3.3, functions in concert with H2A.Z eviction to regulate gene expression during plant thermomorphogenesis	ASF1	[139]
	Water	Drought: Overexpression of OsNAPL6 enhances drought tolerance	OsNAPL6	[140]
	Salt/Osmostress	Salt: OsNAPL6 is recruited to *RAD51 recombinase* (*OsRad51*) promoter, activating its expression and leading to more efficient DNA repair and abrogation of programmed cell death under salinity and genotoxic stress conditions; Mutants of *AtNAP1;3 T* (a member of the NAP family) alter the growth response to ABA and salt	OsNAPL6 and *AtNAP1;3 T*	[140, 141]
	Nutrition	N: Under N deficiency, the *nap1* mutant shows decreased expression of N-responsive marker genes and reduced lateral root growth, indicating that NAP1 is required for normal growth under low nitrogen conditions	NAP1	[142]
	Heavy metal	/		
Biotic stress	Fungus	/		

### Chromatin remodeling

Chromatin, the higher-order organizational form of genetic material in eukaryotic organisms, exhibits significant structural dynamics. Although it maintains the structural integrity of the genome, it participates in various biological processes by precisely regulating gene expression [143]. Dynamic changes in chromatin structure primarily depend on remodeling factors. As key regulatory elements, these factors can modify chromatin accessibility through various molecular mechanisms [144]. From a molecular evolutionary perspective, these highly conserved remodeling factors can be categorized into two functionally complementary groups. One group consists of epigenetic mechanism enzymes that indirectly regulate chromatin states by catalyzing PTMs of histones (e.g. acetylation and methylation), whereas the other group comprises ATP-dependent remodeling complexes that directly utilize energy from ATP hydrolysis to alter histone-DNA interactions. This detailed classification based on mechanisms of action not only reveals the diversity of chromatin remodeling pathways but also provides a crucial theoretical framework for a deeper understanding of gene expression and regulatory networks in eukaryotes. ATP-dependent chromatin remodelers are classified into four distinct families: Switch/sucrose non-fermenting (SWI/SNF), imitation switch (ISWI), chromodomain-helicase-DNA binding (CHD), and INO80 [143].

### Chromatin remodeling is essential for plant growth and development

Chromatin remodeling factors are central regulators of plant growth and development. The SWI/SNF family acts as a multifunctional pioneer of chromatin remodeling, harboring nucleosome sliding, eviction and histone variant exchange activities, and is the only family capable of directly evicting histone octamers. By regulating chromatin accessibility, it participates in processes such as gene transcriptional activation/repression, enhancer function maintenance and chromatin loop formation [145]. The ISWI family is a specialist in nucleosome spacing regulation that achieves precise DNA translocation via the multistate remodeling model, forms a 1 bp DNA bulge during the ATP hydrolysis cycle, and can act on nucleosomes either independently or cooperatively to modulate the dynamic balance between chromatin compaction and decondensation [146]. The CHD family is a modification-dependent precision regulator that recognizes methylated histones via its chromatin domains and binds to DNA through its SANT/SLIDE domains, thereby mediating nucleosome sliding and DNA unwinding. It can cooperate with transcription elongation factors to facilitate the passage of RNA polymerase II through nucleosomal barriers and regulate gene transcriptional elongation [147]. The INO80 family plays a key role in histone variant exchange and damage repair. It recognizes both nucleosomes and hexasomes (which lack one H2A/H2B dimer), adapts to different substrates through conformational rearrangement, and orchestrates the coordination of H2A.Z eviction and H3.3 deposition [148]. The four families exhibit complementary core functions and collectively maintain chromatin homeostasis: SWI/SNF is responsible for chromatin decondensation and histone eviction, creating conditions for gene activation [145]; ISWI sustains uniform nucleosome spacing to ensure chromatin structural stability [146]; CHD is involved in nucleosome remodeling, with certain members playing roles during transcriptional elongation [147]; And INO80 modulates chromatin state switching through histone variant exchange [148]. Together, they synergistically coordinate multiple aspects of chromatin regulation: INO80-mediated H2A.Z eviction often cooperates with SWI/SNF-mediated nucleosome eviction to provide transcriptional activation conditions for stress-responsive genes [149]; The nucleosome spacing maintained by ISWI can be rapidly remodeled by SWI/SNF under stress, enabling rapid switching of gene expression [150]. Additionally, these families participate in diverse developmental processes: BRAHMA and SPLAYED of the SWI/SNF family regulate floral organ development and shoot apical meristem maintenance [151]; ISWI modulates root epidermal cell differentiation and affects cell division rhythm via regulation of cell cycle-related genes [152]; The CHD family is involved in leaf morphogenesis [153]; And INO80 promotes H2A.Z occupancy to regulate cell fate transition in pluripotent stem cells [154].

### Chromatin remodeling confers plant stress resistance via multiple mechanisms

Plants dynamically regulate chromatin accessibility through various epigenetic mechanisms including chromatin remodeling complexes, histone covalent modifications, DNA methylation, histone variant replacement and higher-order chromatin reorganization [155]. This regulation enables the rapid activation of stress-responsive genes and repression of growth-related ones, thereby balancing stress adaptation with growth (Table 8).

**Table 8:** Examples of chromatin remodeling-mediated stress resistance in plants.

**Stress response**		**Theory**	**Genes/transcription factors/proteins**	**References**
Abiotic stress	Illumination	Light: The Phytochrome-interacting factors (PIFs)-INO80 regulatory module facilitates the translation of light signals into chromatin modifications, thereby activating the expression of genes associated with the shading response	PIFs-INO80	[156]
	Temperature	Low temperature: Leaf and flower related (LFR) interacts directly with inducer of CBF expression 1 (ICE1) and activates *ICE1* and *C-repeat binding factor 3* (*CBF3*) genes expression in response to cold stress	LFR	[157]
	Water	Drought: *OsCHR4*, a member of the Snf2 family, enhances drought resistance by increasing wax content in leaf epidermis	*OsCHR4*	[158]
	Oxygen	Oxygen: *RFS* (*Rolled Fine Striped*) encodes a chromatin remodelling ATPase belonging to the CHD3/Mi-2 family and epigenetically regulates genes involved in oxidative stress responses during leaf development by modulating histone H3K4me3 levels, thereby maintaining redox balance	*RFS*	[159]
	Salt/Osmotic stress	Salt: The chromatin remodeling factor CHD3 (PKL) is essential for salt stress tolerance in plants, acting as a positive regulator whose loss of function compromises this trait	PKL	[160]
		Osmotic stress: *The chromatin remodeling factor CHB101* (*ZmCHB101*) mutant exhibits enhanced salt stress sensitivity due to promoter chromatin alterations, resulting in impaired expression of stress response genes	*ZmCHB101*	[161]
	Nutrition	P: *The decrease in DNA methylation 1A* (*ZmDDM1A*) could bind to P- and root development-related genes. Strong associations were found between interacting genes in significantly different chromatin-interaction regions and root traits	*ZmDDM1A*	[162]
		Iron (Fe): Against the backdrop of iron homeostasis, the atypical Histone H2B variant (HTB4) has emerged as a key regulatory factor. *Arabidopsis* HTB4 directly binds to the promoters of Ib subfamily bHLH transcription factors, enhancing their expression by increasing the enrichment of the active chromatin marker H3K4me3	HTB4 and H3K4me	[163]
	Heavy metal	Cd: Cd stress induces global chromatin alterations, with chromatin remodelling playing a key role in activating plant Cd tolerance		[164]
Biotic stress	Bacteria	Pst DC3000: The *Arabidopsis* ISWI chromatin-remodeling factors CHR11 and CHR17, are negative regulators of plant disease resistance	CHR11 and CHR17	[165]

### ncRNA regulation

ncRNAs are cellular RNA molecules that do not encode proteins. These molecules are directly transcribed from DNA and do not participate in protein synthesis. However, some ncRNAs are translated into small peptides that typically contain fewer than 100 amino acids [166]. ncRNAs generally play crucial roles in key biological processes such as gene expression regulation, chromatin modification, and signal transduction [167, 168]. Among these, the well-known rRNA and transfer RNA belong to the category of structural ncRNAs, which are widely present in cells and participate in fundamental cellular activities. Small nuclear RNAs, which are involved in spliceosome formation and the regulation of pre-mRNA splicing, and small nucleolar RNAs, which guide chemical modifications of rRNA and transfer RNA, also belong to the ncRNA category [169–171], Liu et al. has also uncovered the mechanism by which snoRNA promotes protein secretion [172]. In addition, the small RNAs (sRNAs) that range from 18 to 30 nt such as microRNAs (miRNAs) and siRNAs and long ncRNAs (lncRNAs) that exceed 200 nucleotides (nt), which primarily participate in gene expression regulation, are classified as regulatory ncRNAs [169]. Special types include circular RNAs, enhancer RNAs transcribed from enhancer regions to regulate the expression of adjacent genes, and CRISPR RNA, which are used for immune defense [169, 173, 174]. The primary participants in epigenetic regulation are lncRNAs and 24-nt siRNA [167]. Among small ncRNAs, miRNAs and siRNAs are involved in gene silencing. While miRNAs primarily function in post-transcriptional regulation, siRNAs can mediate both post-transcriptional and transcriptional gene silencing (TGS), with heterochromatin-associated siRNAs playing key roles in TGS [168, 175]. These ncRNAs are involved in diverse cellular processes through their complex regulatory networks.

### Regulatory roles of ncRNAs in plant growth and development

The total number of plant lncRNAs accounts for ~80% of all ncRNAs [176]，and play a pivotal role in epigenetic regulation and are essential for plant growth and development. These RNA molecules interact with DNA-binding proteins to specifically recruit chromatin modification complexes to target sites, thereby enabling precise regulation of gene expression [177]. lncRNAs have diverse mechanisms of action in the regulation of plant development. During vernalization, the antisense lncRNA *COOLAIR* (AT5G01675) mediates the conversion of histone marks at the *FLC* locus by activating H3K36me3 to repressive H3K27me3, working synergistically with polycomb repressive complex 2 to achieve epigenetic silencing of the *FLC* gene [178]. The fruit ripening-related lncRNA *FRILAIR* regulates the expression of the laccase gene *LAC11a* by mimicking the target of miR397, thereby influencing the fruit ripening process in strawberries [179]. Complementing the roles of lncRNAs, miRNAs constitute another major layer of post-transcriptional control i.e. central to developmental timing and morphogenesis. The conserved miR156/miR157 family is widely involved in regulating the vegetative-to-reproductive phase transition across angiosperms. In *Arabidopsis*, miR172 regulates somatic embryogenesis, floral development, and flowering time, primarily by modulating key transcription factors such as *wuschel* (*WUS*) and *apetala 2* (*AP2*) [180]. Similarly, in soybean, gma-miR156a orchestrates juvenile development by repressing SQUAMOSA promoter-binding protein-like (SPL) transcription factors, while gma-miR172a influences reproductive development through the downregulation of *AP2* genes [181]. Beyond these conserved pathways, species-specific regulatory modules also exist. For instance, in poplar, leaf growth and development are fine-tuned by the cooperative action of miR164b/e-3p and miR164a-d/e-5p on their *NAC* gene targets [182]. Collectively, these examples underscore the pivotal and diverse roles of ncRNAs in coordinating key developmental transitions. Further elucidation of ncRNA-mediated regulatory networks will provide deeper mechanistic insights and hold promise for strategically improving plant architecture, stress resilience, and ultimately, crop yield.

### Roles of ncRNA in plant stress response

ncRNAs play pivotal regulatory roles in plant responses to various abiotic and biotic stresses, acting as key mediators in the epigenetic and transcriptional regulatory networks that link stress signal perception to adaptive physiological and molecular responses. Taking lncRNAs, miRNAs, circRNA, and siRNAs as the main types, ncRNAs exert their stress-responsive functions through diverse molecular mechanisms [183], which are elaborated as follows (Table 9):

**Table 9:** Examples of ncRNA mediated stress resistance in plants.

**Stress response**		**Theory**	**RNA type**	**References**
Abiotic stress	Illumination	High light: The lncRNA 610 (*MdLNC610*) participates in the regulation of high-light-induced anthocyanin production by functioning as a positive regulator to promote *1-aminocyclopropane-1-carboxylic acid oxidase 1* (*MdACO1*) expression and ethylene biosynthesis	lncRNA	[184]
	Temperature	High temperature: Specific miRNAs regulate high-temperature-induced male sterility in cotton	miRNA	[185]
		Low temperature: In *Arabidopsis*, the two adjacent cold-induced lncRNAs, *SVALKA* and *SVALNA*, finely regulate the key *CBF1* and *CBF3* genes in response to cold stress through distinct mechanisms: *cis-* and *trans-*action, chromatin remodeling, and differential RNA stability	lncRNA	[186]
	Water	Drought: lncRNA2 (*DANA2*) and ethylene response factor 84 (ERF84) co-regulate the expression of *JMJ29* (a gene encoding an H3K9 demethylase), thereby modulating the expression of downstream drought-related genes such as *ERF15* and *galactinol synthase 2*; DRIR, a drought-induced lncRNA, functions as a positive regulator in the plant response to drought stress	lncRNA	[187, 188]
				
	Oxygen	/		
	Salt/Osmostress	Salt: DRIR positively regulates plant adaptation to salt stress	lncRNA	[188]
	Nutrition	N: *TAS3*, known for producing siRNAs that target *auxin response factor* genes, is identified as a nitrogen-responsive lncRNA, with *nitrate transporter 2* predicted as a novel target of *TAS3*-derived siRNAs under low-nitrogen conditions	siRNA	[189]
	Heavy metal	Cd: miRNA-mRNA regulatory networks, including lignin synthesis modules, mediate Cd stress response	miRNA-mRNA	[190]
Biotic stress	Bacteria	Pathogen: The elf18-induced lncRNA *ELENA1* activates plant immunity by binding to the mediator subunit mediator complex 19a and recruiting it to the promoter of the defense gene *pathogenesis-related protein 1*	lncRNA	[191]
	Fungus	*Phytophthora infestans*: lncRNA47980-miR5303-FBA41 module modulates tomato disease resistance by fine-tuning ROS and phytohormone levels	lncRNA47980-miR5303-FBA41	[192]

## The interrelationships between epigenetic mechanisms

The core mechanisms of epigenetics do not operate in isolation. As the Central Dogma dictates, genetic information flows from DNA to RNA and is ultimately translated into proteins [193]. Throughout this process, DNA, RNA, and histones all undergo reversible chemical modifications. It should be noted that ncRNAs are also subject to m^6^A methylation [40]. In plants, RNA polymerase IV (Pol IV) is specifically responsible for the production of a subset of ncRNAs [194]. These transcripts are subsequently processed into 24-nt siRNAs which, via the argonaute RISC catalytic subunit 4 (AGO4) protein, recruit chromatin remodeling factors (such as the CLSY family) and histone modifiers to target loci, leading to the establishment of repressive marks (e.g. H3K9me2) and the induction of DNA methylation. This process is reinforced by a positive feedback loop: Pol V localization depends both on the SUVH2/SUVH9 proteins and on the DNA methylation it helps produce, thereby coupling siRNA production with the maintenance of the methylated state [195].

Furthermore, DNA methylation maintenance is regulated by the ATP-dependent chromatin remodeling factor decrease in DNA methylation 1 (DDM1) [94]. DDM1 also promotes the establishment of H3K9me2. Research in *Arabidopsis* has shown that DDM1 counters the low accessibility caused by nucleosomes containing the histone variant H2A.W by increasing the flexibility of nucleosomal DNA ends. This enables histone methyltransferases to access their target sites and establish the repressive H3K9me2 modification [196].

During transcription, active histone marks such as H3K36me3 and H3K27ac can recruit RNA methyltransferase complexes to nascent RNA, influencing the levels of m^6^A modification in the vicinity. Conversely, m^6^A modifications on transposon RNAs recruit repressive HDACs via their reader proteins, thereby reducing local chromatin accessibility [197]. A regulatory model involving MTA, m^6^A, and pri-miRNA demonstrates that m^6^A RNA methylation regulates miRNA biogenesis [198]. Furthermore, the MTC and the miRNA processing complex (Microprocessor) mediate bidirectional regulatory interactions between miRNA biogenesis and RNA m^6^A modification through protein–protein interactions. Specifically, the MTC, through the SERRATE (SE)-MTB interaction, assists the Microprocessor in recruiting to the miRNA locus, thereby enhancing the efficiency of co-transcriptional processing of pri-miRNAs and ensuring the generation of mature miRNAs; Conversely, the SE protein involved in miRNA biogenesis can activate the m^6^A modification activity of the MTC via phase separation, forming a functional feedback loop [199, 200].

Methylated DNA is recognized by SRA domain-containing proteins (such as the SUVH family), which often possess histone methyltransferase activity and establish repressive histone modifications at these sites. Meanwhile, siRNAs can induce TGS via DNA methylation and mediate histone modifications through DNA methylation-dependent mechanisms [167]. Histone modifications also directly regulate the transcriptional activity of ncRNA genes by altering chromatin states. For example, the *Arabidopsis* HDA6-LDL1/2 complex suppresses lncRNA expression by modulating H3 acetylation and H3K4me2 levels [201].

A direct bidirectional feedback loop between DNA methylation and m^6^A has been identified in plants. During tomato fruit ripening, the m^6^A demethylase SlALKBH2 relies on ethylene insensitive 2 (SlEIN2) to promote the expression of the DNA demethylase SlDML2, with SlEIN2 regulating *SlDML2* expression post-transcriptionally [202]. Additionally, oxidative modifications of SlALKBH2 enhance its regulatory effect on the *SlDML2* transcript [203]. Concurrently, SlALKBH2 itself is regulated by DNA methylation; Specifically, DNA methylation influences mRNA m^6^A methylation by targeting SlALKBH2, thereby modulating fruit ripening. This forms a molecular circuit in which DNA methylation and m^6^A modifications mutually regulate fruit ripening [204].

**Figure 7:**
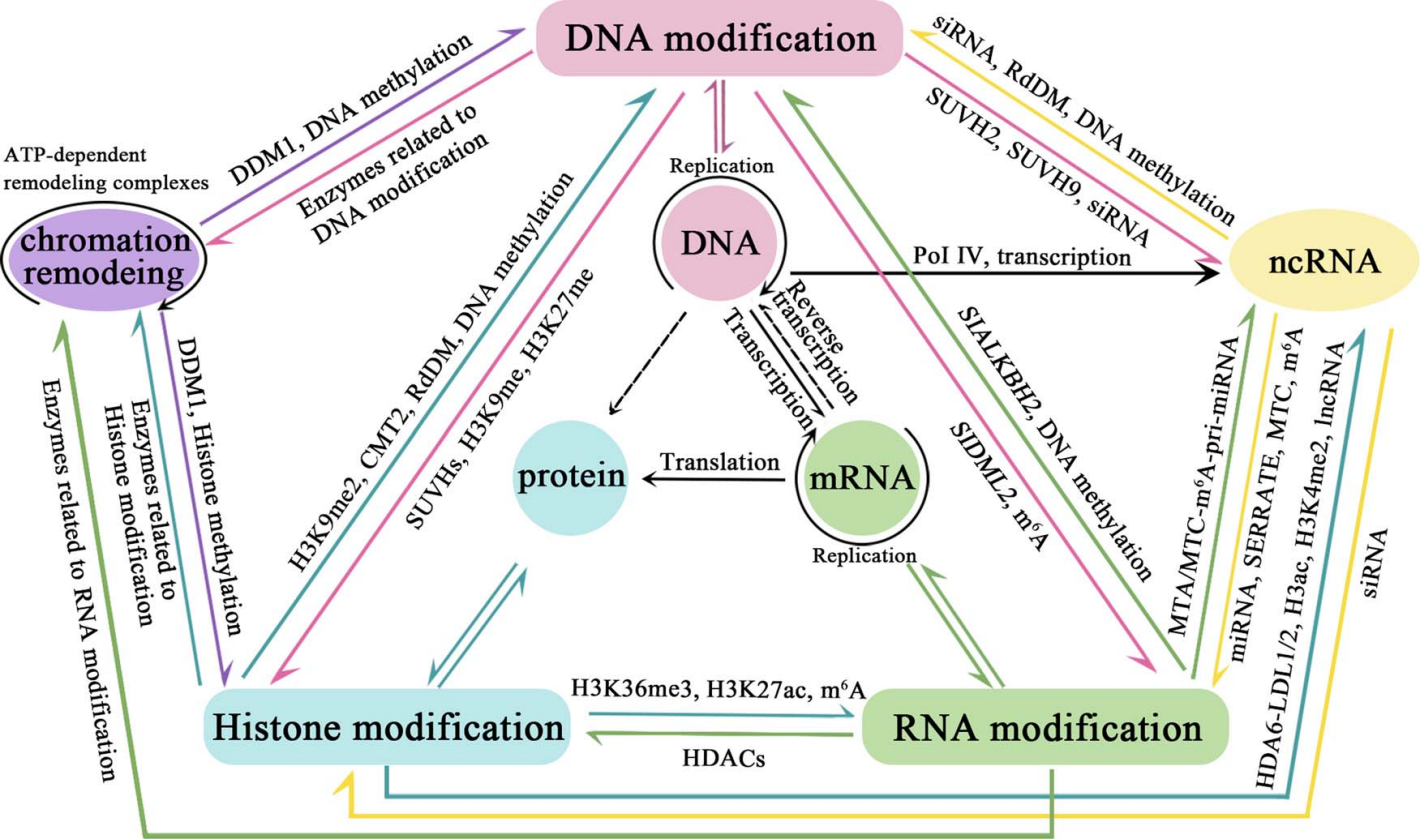
Epigenetic regulatory networks in plants: Synergistic interactions between DNA modifications, histone modifications, RNA modifications, ncRNA and chromatin remodelling

The interrelationships among the aforementioned epigenetic mechanisms are illustrated in Fig. 7. The deeper and broader interactions underlying these epigenetic networks remain to be further explored and elucidated.

### Transgenerational inheritance of epigenetic mechanisms

Plants exhibit remarkable environmental adaptability and can transmit environmentally induced adaptive traits to subsequent generations through epigenetic mechanisms [205]. However, direct molecular genetic evidence of this phenomenon is lacking. Song et al. made a breakthrough in their study on the adaptive traits of rice cultivated in the northern regions [206]. They systematically analyzed the epigenetic evolutionary patterns of rice under low-temperature stress. Their study provides the first molecular-level confirmation that environmentally induced epigenetic variations can be stably inherited and mediate the transgenerational expression of adaptive traits. This significant discovery not only offers direct evidence for the epigenetic regulatory mechanisms underlying plant environmental adaptability but also provides robust molecular genetic support for Lamarck’s theory of ‘acquired inheritance’.

Plants achieve the intergenerational transmission of environmental adaptability through precise epigenetic mechanisms that regulate gene expression. Methylation levels are dynamic and vary during development. Plants with long-term stress resistance can stably inherit stress-related methylation memory via mitosis or meiosis, thereby preserving the epigenetic memory of their cellular precursors and enabling subsequent generations to exhibit enhanced stress tolerance [207]. A direct relationship between environmental perception, epigenetic remodeling, and memory has been demonstrated in plants [208]. For example, plastids and mitochondria can detect changes in signaling molecules that induce epigenetic mechanisms in the nuclear genome through retrograde signal transduction. Under abiotic stress conditions, the expression of MutS Homolog 1 (*MSH1)*, a gene involved in maintaining plastid genome integrity, is downregulated. Inhibition of *MSH1* generates plastid-derived stress signals that trigger nuclear events, including genome-wide regulation of proximal DNA methylation of TEs, mediated by histone deacetylase 6 and MET1 [209]. These events regulate the expression of clock-, hormone-, and stress-related genes, collectively conferring cross-representative plasticity in plants. Plants respond to changes in light through photoreceptors and light signaling components. Adaptation to light and heat involves extensive transcriptional reprogramming, primarily mediated by nuclear structural changes [210], such as chromatin remodeling. This remodeling includes the incorporation of histone variants [156] and HPTMs, such as H2B monoubiquitination [211] and histone acetylation [212], as well as alterations in DNA methylation patterns [213]. Heat sensing exemplifies plant domestication, involving the epigenetic regulation of heat shock factors and heat morphogenesis genes through modifications in histone marks and histone variant deposition [214]. In *Arabidopsis*, flowering time is regulated via epigenetic switches, with vernalization memory stably inherited during female gamete formation, and exhibiting maternal transmission characteristics [215]. A study has also shown that specific low-methylation patterns in centromere-adjacent regions confer both disease resistance and tolerance to abiotic stress [216], revealing synergistic epigenetic regulatory mechanisms in response to multiple stresses.

### Long-term stress memory during asexual reproduction

Epigenetic information can be transmitted through somatic inheritance and asexual reproduction (clonal reproduction) during mitosis via stem cells located in the meristematic tissues [214]. During mitosis, specific HPTMs are required, such as chromatin-associated histone H3S10p and other mitosis-specific HPTMs [217]. Domains enriched with repressive histone marks (e.g. H3K27me3) are stably maintained throughout mitosis. Studies have demonstrated that the *Arabidopsis FLC* gene gradually accumulates the repressive histone mark H3K27me3 during vernalization. This epigenetic mechanism achieves a stable inheritance through two key mechanisms. First, it is retained during mitosis via replication-coupled processes. Second, during DNA replication, the methylated histone variant H3.1 is specifically incorporated into newly formed chromatin, ensuring the stable maintenance of H3K27me3 modification throughout the S phase [218, 219]. Extensive experimental evidence has indicated that asexual reproductive systems possess unique epigenetic and intergenerational memory capabilities. For example, *Arabidopsis* plant regenerated *in vitro* can retain the epigenetic characteristics and physiological traits of their parental tissues, demonstrating that clonal lineages can stably transmit epigenetic states [220]. Asexual propagation methods, such as vegetative reproduction (e.g. through fragmentation), have a greater capacity for transmitting epigenetic memory than sexual reproduction [221]. This is because they bypass the epigenetic reset mechanisms that occur during gametogenesis and early embryogenesis. Consequently, they maintain epigenetic states more stably, in contrast to the partial epigenetic reset often observed in sexual reproduction.

### Intergenerational and transrepresentational epigenetics after sexual reproduction

Since the late 20th century, extensive research has demonstrated that epigenetic information can be transmitted across generations through sexual reproduction [222]. Researchers have used the *Arabidopsis* epigenetic recombination inbred line, a unique genetic resource, to provide conclusive experimental evidence supporting epigenetic inheritance across generations [223, 224]. Various epigenetic mechanisms, including DNA methylation, sRNAs, and histone modifications, are involved in this complex process [36, 214]. Many of these mechanisms are also key mediators of long-term stress memory in asexual reproduction, although their stability and transmission patterns differ significantly between sexual and asexual modes. However, the specific molecular mechanisms underlying the transgenerational transmission of epigenetic information, such as the synergistic interactions among different modifications and epigenetic reprogramming during gamete formation, remain the subject of considerable debate and unresolved questions within the academic community.

Research has demonstrated that parental DNA methylation patterns can be partially retained in germ cells and transmitted to offspring, thereby regulating gene expression in subsequent generations. In *Arabidopsis*, DNA methylation changes induced by osmotic stress, the space environment, and γ-ray radiation have all been shown to undergo transgenerational inheritance [36, 225, 226]. This process involves siRNA-mediated epigenetic regulatory mechanisms. In the male germline, siRNAs derived from nutritive cells enhance sperm DNA methylation, whereas siRNAs derived from central cells may perform similar functions in oocytes [214]. In grasses, wheat miRNAs play a role in regulating offspring processes, such as ROS scavenging, hormone signaling, and carbon fixation. In rice, lncRNAs regulate the memory genes related to hormone synthesis [227]. Notably, heat stress-induced reductions in H3K27me3 levels and *trans-*acting siRNA synthesis promote the transgenerational inheritance of traits such as early flowering (ELF8) and immune regulation [228]. However, the role of histone modification in transgenerational epigenetics remains controversial. Due to the extensive reprogramming of histone modifications during germ cell development, parental histone modification states often fail to be stably inherited [227]. The core of this controversy is not whether histone modifications are involved in transgenerational regulation, but whether specific histone modifications can act as independent and stably heritable transgenerational epigenetic carriers. This controversy is particularly prominent in studies related to plant stress memory and developmental regulation. Several studies have uncovered the molecular mechanisms underlying the maternal inheritance of the ‘winter cold memory’ (vernalization effect) in *Arabidopsis*. Specifically, the repressive histone mark H3K27me3 is established at the *FLC* locus during prolonged cold exposure, stably retained in egg cells, and transmitted to the zygote and early embryos via the female germline [215]. Consequently, progeny maintain *FLC* silencing without experiencing low temperature, enabling the transgenerational regulation of flowering time. However, accumulating evidence has challenged the direct transgenerational inheritance of histone modifications. Inflorescence marker line analysis in *Arabidopsis* detected H3K27me3 in microspores, indicating its transmission from microsporocytes through meiosis. In addition to H3.3, sperm chromatin incorporates the sperm-specific histone variant H3.10, which cannot be methylated at the K27 residue. Furthermore, during sperm development, components of polycomb repressive complex 2 (PRC2) (including subunits encoding histone methyltransferases) and most subunits of PRC1 are undetectable, leading to a global loss of H3K27me3 on histone-based sperm chromatin [229]. These findings reveal the complex regulatory network underlying transgenerational epigenetics in plants and highlight the significant differences in genetic stability among the various modification mechanisms.

## Conclusions and perspectives

Epigenetic regulation is central to plant growth, development, and adaptation [3]. Its unique characteristics of intergenerational inheritance provide a crucial mechanism for plant populations to adapt to changing environments. The core feature of plant epigenetic regulatory networks is the cross-collaboration of different modification mechanisms, which is the key to the precise response of plants to developmental signals and environmental stresses. However, the synergistic regulatory networks among these modifications and their integrative mechanisms in forming intergenerational memory have yet to be systematically explored. This fragmentation of knowledge underscores the need for more in-depth and systematic research, particularly addressing key scientific questions such as the specific transmission of epigenetic information, the epigenetic decoding of environmental signals, and the molecular basis of epigenetic memory.

Rapid advancements in molecular biology, technologies, and equipment have created unprecedented opportunities in plant epigenetic research, also opened new avenues for in-depth exploration of epigenetic regulatory mechanisms. For example, the application of single-cell sequencing technologies, enables the analysis of epigenetic mechanism heterogeneity at a single-cell resolution [230, 231]. The development of ultra-high-resolution microscopy techniques, such as super-resolution localization microscopy and super-resolving light microscopy, has enabled the real-time observation of the spatial distribution of epigenetic mechanisms within the cell nucleus at the nanoscale [232]. These technological innovations will enable a deeper understanding of the dynamic processes of epigenetic mechanisms and will facilitate the development of spatiotemporally precise epigenetic editing tools. Thereby contributing to more resilient agricultural systems. Looking ahead, we can leverage epigenetics to directly optimize the metabolic strategies that crops employ in response to environmental fluctuations. By precisely modulating epigenetic switches within stress-responsive metabolic pathways, we can enhance the crop's buffering capacity against variable conditions. The most transformative direction lies in directed epigenetic breeding. By integrating multi-omics approaches, we can systematically decipher the core epigenetic networks governing crop stress resistance. Using CRISPR-based epigenome editing tools, we can then precisely remodel these regulatory patterns, thus enabling directed epigenetic breeding.

In conclusion, integrating basic epigenetic research with translational agricultural applications will drive crop breeding innovation and enhance climate resilience. Addressing these unresolved challenges will enable epigenetics to become a core force in safeguarding global food security and promoting sustainable agriculture amid rapid climate change.
